# The Neuroprotective Role of Exercise in Alzheimer’s Disease: An Integrative Review of Animal and Human Studies

**DOI:** 10.3390/neurolint18060113

**Published:** 2026-06-08

**Authors:** Danqing Xiao, Akshita Duvvuri, Lenna V. Makrigiannis, Catherine Fuller

**Affiliations:** 1 Department of STEM, School of Arts, Sciences, and Education, Regis College, Weston, MA 02493, USA; 2 McLean Imaging Center, McLean Hospital, Harvard Medical School, Belmont, MA 02478, USA; 3 Department of Neuroscience, School of Health Sciences, Regis College, Weston, MA 02493, USA; akshitaduv@gmail.com; 4 School of Health Sciences, Regis College, Weston, MA 02493, USA

**Keywords:** Alzheimer disease, exercise, neuroprotection, cognition, BDNF, neuroinflammation, mitochondrial function, Tai Chi

## Abstract

Alzheimer’s disease (AD), the leading cause of dementia, is characterized by progressive cognitive decline along with hallmark brain pathologies including amyloid-beta accumulation, hyperphosphorylated tau, neuroinflammation and neuronal mitochondrial dysfunction. As current pharmaceutical treatments only provide modest symptomatic improvement, there is an urgent need for effective non-pharmaceutical treatment options for the prevention or slowing down of this disease. This review synthesizes results from randomized controlled trials, observational studies, and animal model research on the ability of exercise to influence cognitive functions, brain structural changes, inflammatory processes, and neuroplasticity-related pathways. Exercise has demonstrated the capacity to enhance neurotrophic signaling, improve the regulation of mitochondria, improve cerebrovascular function and reduce pro-inflammatory cytokine levels in preclinical and mild cognitive impairment (MCI) subjects. Additionally, aerobic and resistance training has been shown to enhance physical performance and functional capacity. Furthermore, mind–body, dual-task and multimodal types of interventions may also provide additional cognitive and psychological benefits. Although the overall cognitive effect of exercise in individuals with established AD is generally small, it has been demonstrated that exercise can contribute to maintaining brain health through multiple interconnected metabolic, vascular and molecular pathways, thereby preserving cognitive reserve and slowing disease progression, particularly when initiated during early to midlife prior to the onset of AD symptoms. Therefore, future research will require establishing stage-specific exercise recommendations based on modality type, intensity and duration to achieve optimal clinical outcomes.

## 1. Introduction

Alzheimer’s disease (AD), the leading cause of dementia worldwide, affected an estimated 7.2 million Americans aged 65 and older in 2025, with global dementia cases exceeding 57 million (primarily in low- and middle-income countries) and projections indicating a rise to 139 million by 2050 unless effective prevention strategies are implemented [1]. AD manifests as progressive cognitive decline—particularly in memory, executive function, and activities of daily living—driven by hallmark pathologies: amyloid-β (Aβ) plaque accumulation, tau hyperphosphorylation forming neurofibrillary tangles, chronic neuroinflammation, mitochondrial dysfunction, synaptic loss, and eventual neuronal death [2,3,4]. The prevalence of AD increases vastly with age and in 2024 it was ranked the fifth leading cause of death among Americans aged 65 and older [5].

Despite advances in pharmacotherapy, including cholinesterase inhibitors, memantine, and recently approved anti-amyloid monoclonal antibodies (e.g., lecanemab, donanemab), treatments remain largely symptomatic or offer only modest slowing of progression in early stages, with limited disease-modifying impact, accessibility challenges, and potential side effects such as amyloid-related imaging abnormalities (ARIAs) [6]. This underscores the urgent need for accessible, low-risk, non-pharmacological interventions to delay onset, slow progression, or preserve function across the AD continuum.

Physical activity emerges as one of the most promising modifiable lifestyle factors that may prevent or delay onset of the disease [7,8,9,10]. Longitudinal and epidemiological studies consistently associate moderate-to-vigorous physical activity in mid and late life with reduced AD incidence and slower cognitive decline, contributing to the growing recognition of physical inactivity as a key modifiable risk factor [11]. Mechanistically, exercise exerts multi-level neuroprotective effects, including increased hippocampal volume, enhanced cerebral blood flow, promotion of neurogenesis, and strengthened synaptic plasticity—processes that build cognitive reserve and counteract AD vulnerability [12,13]. Exercise influences gene expression supporting neuronal resilience such as brain-derived neurotrophic factor (BDNF) upregulation, inflammatory pathways, mitochondrial dynamics and biogenesis, cerebrovascular health, and even the gut–brain axis [14,15,16,17,18,19,20]. For instance, exercise modulates gut microbiota composition, increases short-chain fatty acid production, reduces pro-inflammatory species, enhances blood–brain barrier integrity, supports microglial Aβ clearance, and improves neuronal energy metabolism [18].

Clinically, exercise improves executive function, episodic memory, balance, quality of life, and functional independence in at-risk or symptomatic older adults [10]. Randomized controlled trials (RCTs) demonstrate that aerobic and resistance training preserve hippocampal volume and cardiorespiratory fitness in mild cognitive impairment (MCI) and early AD, though global cognitive improvements are often modest or inconsistent [21]. Emerging evidence supports additional benefits from mind–body modalities (e.g., Tai Chi, yoga) on executive function, emotion regulation, neural connectivity, and stress reduction [19,22,23]. Multidomain interventions incorporating exercise, diet, and stress management further suggest potential to slow progression in early stages of dementia [24].

Collectively, these findings position structured physical activity as a multi-target neuroprotective strategy acting across metabolic, vascular, inflammatory, and systemic pathways relevant to AD pathology. However, heterogeneity in modalities, intensities, timing, and disease stage limits direct comparisons. Integrative syntheses combining human RCTs, observational data, animal mechanistic studies, and emerging pathways (e.g., gut–brain axis) remain necessary to inform optimized prescriptions. The most recent review shows that minimum physical activity with at least 15 min at a time at least three times a week is beneficial to reduce the risk of developing AD. However, any form of physical activity appears protective including household chores [10].

This integrative review synthesizes evidence from RCTs, observational cohorts, animal models, and mechanistic investigations to evaluate the neuroprotective role of exercise in AD, with emphasis on cognitive/structural outcomes, underlying biological mechanisms, the critical influence of intervention timing (particularly early/preclinical stages), and implications for stage-specific exercise recommendations to maximize neuronal resilience and potentially slow disease progression.

### 1.1. Positioning in Relation to the Recent Literature

A systematic search conducted in April 2026 identified approximately 75 review articles using the keywords “exercise” and “Alzheimer’s disease.” After excluding reviews focused solely on biomarkers, diagnostic scales, motor symptoms without cognitive outcomes, non-exercise non-pharmacological devices, sleep, mental health in isolation, or other primary neurodegenerative conditions, approximately 30 highly relevant reviews were retained. These reviews predominantly address isolated mechanisms or examine exercise across multiple neurodegenerative diseases.

Collectively, these works underscore that cognitive function in Alzheimer’s disease is fundamentally shaped by the neuro-nutritional–metabolic axis, which governs nutrient homeostasis, metabolic efficiency, and the clearance of neurotoxic waste. Physical exercise emerges as a central facilitator of nutrient utilization, cellular and systemic homeostasis, and brain waste removal. Key recent insights at the molecular level include: (1) lipid and glucose homeostasis, with exercise enhancing insulin sensitivity in the context of AD as a “type 3 diabetes” metabolic disorder [25,26]; (2) inter-organ axes, particularly muscle–brain (myokine-mediated); [27,28], gut–microbiota–brain [29], liver–brain [30], and spleen–brain [31] signaling pathways that transduce peripheral exercise signals into central neuroprotection or removing misfolded protein aggregates by autophagy; (3) the role of iron homeostasis in exercise-mediated slowing of cellular senescence and its tight linkage to mitochondrial function [32,33].

**Figure 1:**
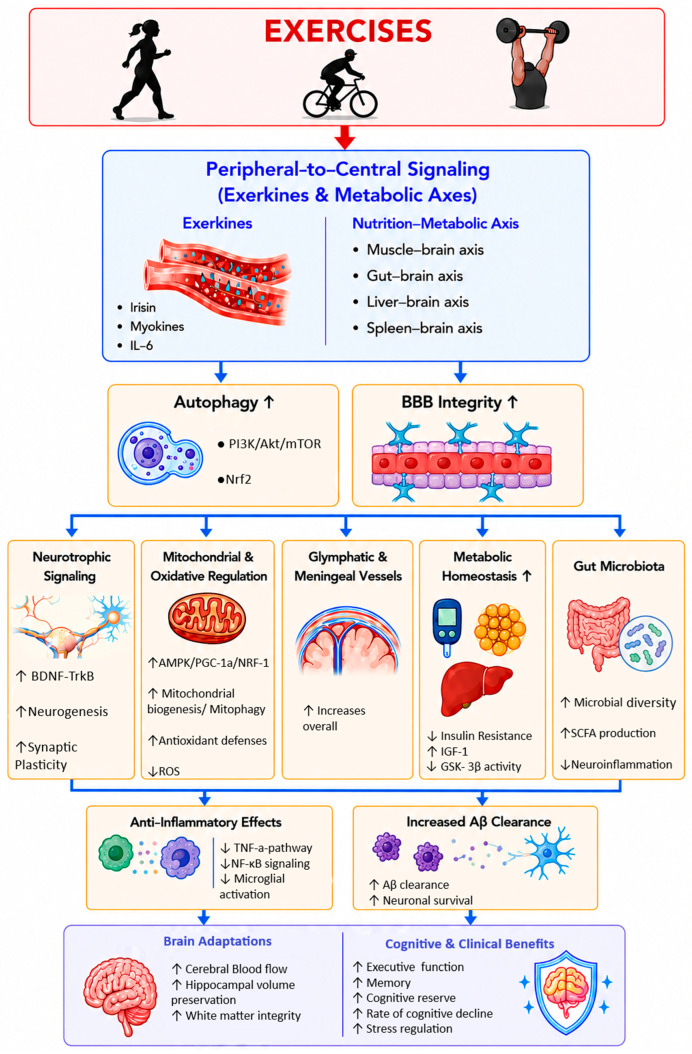
Multi-level neuroprotective mechanisms of exercise-mediated reversal of age-related neurodegenerative processes associated with Alzheimer’s disease.

At the organelle level, recent reviews highlight exercise-induced mitigation of mitochondrial dysfunction [34], endoplasmic reticulum stress [35], and activation of autophagy via irisin and related myokines, which together support neuroplasticity and cognition [9,36,37,38]. Cellular-level effects include favorable modulation of astrocyte and microglial (polarization) phenotypes with enhanced phagocytic clearance [39]. Organ- and organ system-level mechanisms encompass strengthened CNS–peripheral immune crosstalk (see above the inter-organ axes), glymphatic/meningeal lymphatic clearance of amyloid, cardiovascular benefits, and improved sleep patterns (see Section 4.3). Finally, exercise synergizes with lifestyle changes (such as diet) to promote hippocampal neurogenesis, cognitive reserve, and resilience via Nrf2 signaling and hepatokine release [30,36,37,40,41] (See Figure 1).

### 1.2. Distinction of the Present Review

While excellent recent syntheses, such as the narrative review by Brendborg and Febbraio [42], elegantly summarize five core neuroprotective mechanisms of exercise in AD (anti-inflammatory effects, enhanced Aβ clearance, hippocampal neurogenesis, cognitive resilience and reserve), the current integrative review extends beyond this by:

1. Systematically interconnecting effects across multiple biological scales (molecular → organelle → cellular → organ → organ-system) and their overlapping signaling pathways.
2. Providing a balanced synthesis of both human clinical trials/RCTs and transgenic rodent AD models, emphasizing translational relevance and timing of intervention.
3. Incorporating mind–body (Tai Chi, yoga, dancing), dual-task, aerobic, resistance, and multimodal modalities with modality-specific mechanistic insights.
4. Highlighting emerging gut–brain, muscle–brain, and other inter-organ axes as integrative hubs that link peripheral exercise adaptations to central AD pathology.
5. Particularly, it delivers a detailed comparison of different exercise forms—including aerobic, resistance, mind–body, dual-task/multimodal, and multidomain interventions that combine physical activity with cognitive training—and evaluates their differential impacts on cognitive outcomes, neuroplasticity, and AD-related pathology.

Thus, this review offers a more comprehensive, multi-level framework that not only summarizes but actively integrates disparate mechanistic streams, reinforcing exercise as a practical, multi-target neuroprotective strategy across the AD continuum.

## 2. Neuroprotective Role of Exercise in Animal and Human Studies

### 2.1. Findings in Animal Studies

In animal models of AD, particularly transgenic rodents recapitulating amyloid-β and/or tau pathologies (e.g., APP/PS1 mice), exercise interventions—aerobic modalities such as treadmill running, swimming, or voluntary wheel running and anerobic resistance exercise such as climbing stairs with added loads to the tail—consistently demonstrate neuroprotective effects. Examples are summarized in Table 1; these studies reveal reductions in amyloid-β deposition and total tau, attenuation of neuroinflammation, elevations in neurotrophic factors such as BDNF, and improvements in synaptic plasticity markers, collectively contributing to better hippocampal function, spatial learning and memory performance [43,44,45,46,47,48,49,50]. Further, predominantly aerobic modalities reveal enhancements in mitochondrial function (biogenesis and mitophagy) and white matter volume and reduced amyloid-β in the hippocampus and cortex [51,52].

Resistance exercises in AD animal models reveal enhancements in muscle size, grip strength, serum insulin-like growth factor-1 (IGF-1), nerve growth factor (NGF) and NT3 (neurotrophin-3), and tropomyosin receptor kinase (Trk) A and B and clearing up the hippocampal Aβ burden, through reducing microglia activation, with or without increasing BDNF and inconsistent changes in hippocampal inflammatory interleukin cytokines [27,47,49,50]. In most cases, pro-inflammatory cytokines are reduced and anti-inflammatory cytokines are enhanced, except interleukin-6 (IL-6), which is released after exercise and has multiple functions in muscle and neuroprotection. In long-term exercise, baseline levels of IL-6 level are lowered and the same with inflammatory markers such as tumor-necrosis factor-alpha (TNF-α) [27,46,48]. Combined exercise with alternating weeks of aerobic and resistance exercise seems to have added benefit; for example, increased NGF is only found in combined exercise [49].

Mechanistically, exercise modulates key pathways implicated in AD pathogenesis. It restores redox homeostasis via upregulation of protein translation in the hippocampus tissue, and protects synapses from oxidative damage while supporting mitochondrial integrity [46,49,53]. Exercise further enhance amyloid clearance and glymphatic function through astrocyte-mediated mechanisms ameliorating circadian disruption in AD models [54]. These preclinical findings clarify the molecular and cellular mechanisms, neurotrophic, anti-inflammatory, mitochondrial, and exerkine-mediated, underlying exercise’s neuroprotective effects and inform translation to human studies [55,56]. Importantly, animal data underscore that exercise efficacy is timing-dependent: interventions yield maximal benefits when initiated early, prior to substantial neuropathological burden (e.g., pre-plaque accumulation or mild pathology stages).

**Table 1:** Neuroprotective role of exercise in animal studies.

Study	Model Used	Age (Months)	Design	Results
[57,58]	Male rats	2	Up to 12 weeks wheel-running	↑ Neurotrophic level and signaling (BDNF), improvement in memory task
[51]	APP/PS1 male and female mice	6	4 months treadmill running exercise, 5x/week	↑ White matter volume, ↑spatial learning and memory abilities in female mice
[59,60]	APP/PS1-AD male mice, mixed gender respectively	6	4 weeks–4 months resistance exercise	↓ Plasma corticosterone, ↑ microglial cells around plaques, ↑BDNF ↑cognitive outcomes
[56]	Amnestic mice	2	4 weeks treadmill running	↓ Neuroinflammation; improved cognition and memory
[45]	APP/PS1-AD mice (gender not specified)	3	12 weeks treadmill exercise	↑ Mitochondrial biogenesis, mitophagy; ↑ synaptic markers ↑ memory
[61]	Male and female AD rates (treated with STZ)	3 (11–12 weeks old)	12 weeks treadmill exercise 5x/week	↑ Hippocampal BDNF in females not males
[62]	Male rat AD model	2	4 weeks aerobics training	↑ Synaptic plasticity and recognition memory
[49]	Female ovariectomized, D-Gal Wistar AD rats	6–7	6 weeks, resistance, aerobic, or combined resistance and aerobic exercise, 3x/week	Muscle hypertrophy (soleus and flexor digitorum brevis) ↑ Cognition ↓ Hippocampal and cortical Aβ and oxidative stress ↑ Serum IGF-1 ↑ Hipp IGF-1 only in aerobic exercise and NGF only in combined exercise. No change in BDNF
[47]	Male 3xTg AD mice	3	9 weeks of resistance and aerobic exercise 3x/week	Muscle hypertrophy (gastrocnemius) ↑ Grip strength ↓ Hippocampal and cortex Aβ ↑ Serum IGF-1
[46]	Male 3xTg AD mice	9	4 weeks of resistance exercise 3x/week	↑ Cognitive function ↓ Hipp and cortex Aβ, total tau, TNF-α (FC), IL-1β (liver, serum) ↑ Pro-inflammatory IL-6 (FC, hipp) cytokine, PGC-1 (hipp) ↓ Microglia and astrocytes activation;
[48]	Male APP/PS1 (C57Bl/6)	6–7	4 weeks of resistance exercise 5x/week	↑ Motor activity ↓ Hipp Aβ plaques, ↓ hippocampal pro-inflammatory IL-6, IL-4, IL-1a cytokine
[50]	Male Wistar AD rats (treated with STZ)	adult	8 weeks resistance exercise 3x/week	↑ Learning and memory, ↑ Hippocampal neurotrophins (BDNF, NGF, NT3), and Trk [49] (A and B) receptors

Notes: BDNF, brain-derived neurotropic factor; FC, frontal cortex; Hipp, hippocampus; IL, interleukin; IGF, insulin-like growth factor; NGF, nerve growth factor; NT3, neurotrophin-3; PGC-1, peroxisome proliferator-activated receptor gamma coactivator-1; STZ, streptozotocin; TNF-α, tumor necrosis factor-α. ↑, increase; ↓, decrease.

AD hallmarks [31]: This principle of earlier intervention is explored further in Section 2.3 in relation to both animal and human evidence (Table 2).

Overall, emerging evidence highlights that exercise induces the release of circulating substances called exerkines, such as myokines, BDNF and other bioactive molecules, which reduce oxidative stress, modulate inflammation, preserve mitochondrial function, and promote neuroplasticity, further preventing muscle wasting and cognitive decline across neurodegenerative models [27,46,55,63].

### 2.2. Findings in Human Studies

Table 2 summarizes key examples of randomized controlled trials (RCTs) and intervention studies examining the effects of structured exercise across the AD spectrum, from cognitively normal older adults to those with mild–moderate AD. These studies predominantly feature aerobic, resistance, multicomponent or multimodal, or mind–body modalities (e.g., Tai Chi), with durations ranging from 12 weeks to 4 years.

In healthy older adults (Table 2A), aerobic exercise interventions for one year produced structural and functional brain benefits, including increased hippocampal volume and spatial memory [64] and enhanced functional connectivity between temporal cortices [65]. In multicomponent combination training combining aerobic exercise, resistance exercise with mindfulness training at 6 months or 4 years did not demonstrate significant improvement in memory or cognition in older adults, except when aerobic exercise is combined with a healthy diet [66,67]. However, a survey-based study with randomly sampled participants reported that physical exercise combined with computer use was associated with reduced odds of developing MCI [68]. Moderate–vigorous physical activities do show the exercise benefit in preserving cortical thickness with reduced amyloid burden in middle-aged adults at risk for AD [69]. An umbrella review of existing meta-analyses of RCTs shows strong evidence of a protective effect of regular exercise in reducing AD risk [16]. Perhaps aerobic exercise with longer duration and higher intensity would be efficacious to garner the exercise benefit, and the literature is inconsistent in suggesting that each type of exercise, aerobic, resistance or all forms of multicomponent exercise, delivers improvement in global cognition [67,70,71]. In fact, it is aerobic exercise (at least 12 weeks) not resistance exercise that significantly improved verbal working memory and spatial memory in older adults with probable mild cognitive impairment (MCI) [72,73]. On the other hand, for older adults especially when their frailty is increased, resistance exercise and balance training on gait are strongly recommended as these training modalities improve muscle strength and coordination, motor performance and quality of life in general [74]. Overall, a recent review shows that all forms of exercise with minimum requirements are protective against developing AD in late life [10].

Meta-analysis of clinical trials confirmed that exercise increases global cognition and MMSE scores, executive function, memory and attention in MCI and AD [75,76]. The strongest cognitive benefits are found in individuals with MCI, who participate in moderate-intensity exercise, at least 30 min duration per session and over three times per week, with a total duration of at least 12 weeks [76,77]. Multiple RCTs demonstrated improvements in functional brain connectivity of the default-mode network between the hippocampus and angular gyrus and less hippocampus loss and brain atrophy, delaying global cognitive decline measured by the Alzheimer’s Disease Assessment Scale–Cognition (ADAS-Cog), supplemented with tests of executive function, following (moderate–high-intensity) aerobic exercise [78], high-intensity resistance [74,79,80,81], multicomponent [82,83], resistance-only [82,84,85] programs. Larger RCTs such as SYNERGIC (20 weeks aerobic + resistance) and EXERT (12–18 months moderate aerobic) in sedentary older adults with amnestic MCI showed beneficial effects of moderate–high intensity aerobic exercise alone, or combined with cognitive training and/or lower-intensity stretching/balance activities and resistance exercise in global cognitive stabilization, preserving hippocampal and its subfields over 6–12 months and preventing cognitive decline [78,80,86] (Table 2B). When compared to matched usual-care cohorts (e.g., from ADNI), both exercise arms showed significantly less cognitive decline, suggesting a stabilizing rather than intensity-dependent disease-modifying effect in early-stage risk. Although a multidomain intervention (combination of different types of exercise and cognitive training and diet) did not prevent the cognitive decline in MCI in a Japanese cohort or add additional benefit in MCI in a Chinese cohort [87,88], a recent review shows that cognitive stimulation training alone has various benefits including improved cognitive function for mild and moderate dementia patients, and it is practiced in Australian communities [89]. Mindfulness exercise interventions, such as Tai Chi Chuan lasting at least 36 weeks, are more effective than fitness walking in improving cognition among individuals with both MCI and type 2 diabetes [90].

In mild–moderate AD, either aerobic exercise or multicomponent training significantly improved functional independence (Barthel Index), physical performance (6 min walk test), and neuropsychiatric symptoms such as depression. However, cognitive effects were inconsistent. Clinical trials showed no possible or significant cognitive benefit [91,92,93,94,95]. Notably, the largest DAPA trial in England (Table 2) reported no cognitive benefit with a higher ADAS-cog score and slightly lower quality-adjusted life year (QALY) in the moderate–hard exercise group versus controls [96,97,98], though physical (aerobic) fitness and functional independence or capacities outcomes (e.g., mobility) often improved or were preserved [94,98,99,100]. An umbrella review of existing meta-analyses of RCTs confirmed thatphysical exercise seems to improve global cognition, physical performance such as functional reach, balance and 6 min walking test, and functional independence in patients with AD [14,16]. Across AD stages, exercise reliably enhanced physical performance, gait, mobility, balance, and self-efficacy, even when global cognition remained unchanged.

Overall, human trials demonstrate robust peripheral and functional benefits from exercise, with cognitive preservation or improvement most evident when initiated in preclinical or MCI phases. Global cognitive effects diminish in mild–moderate AD, where interventions primarily support functional reserve, mobility, and quality of life. These patterns reinforce the importance of early intervention and highlight the need for personalized prescriptions to maximize neuroprotective potential. Mechanistic insights from animal models (see Section 2.1) provide complementary support for these translational observations.

**Table 2:** Human exercise trials across the cognitive normal and Alzheimer’s disease spectrum.

**A. Healthy Older Adults**					
**Study (Examples)**	**N**	**Population**	**Duration**	**Exercise Type**	**Key Outcomes**
[64]	120	Older adults	12 months	Aerobic walking	↑ Hippocampal volume, spatial memory, ↑ BDNF
[65]	65	Older adults	12 months	Aerobic walking	↑ Temporal lobe connectivity, in link to ↑ BDNF and VEGF
[101]	63	Older women with subjective memory decline and CVD risk	12 weeks	Cognitive mind–body modalities (yoga or MET)	↑ Hippocampal subregion connectivity
[66]	585	Older adults	6 months	Multicomponent exercise (mindfulness, aerobic and resistance training)	No change in episodic memory and cognition
DR’s EXTRA [67]	1401	Older adults	4 years	Different types	No change in cognition, except combining moderate-intensity aerobic exercise and a healthy diet
ALFA Study [69]	337	Middle-aged adults (45–65)	4 years	Moderate/vigorous activities	↑ Cortical thickness; ↓ dose-dependent amyloid burden
[102]; [103]	1967	Middle-aged and older (more women)	Multi-years	Square dance	↑ Cognition, hippocampal volume, mental health
**B. Mild Cognitive Impairment (MCI)**					
**Study (Examples)**	**N**	**Population/Stage**	**Duration**	**Exercise Type**	**Key Outcomes**
[78]	296	Amnestic MCI	6 months	Moderate–high aerobic	Preserved cognition and hippocampal volume without decline
[81]	100	MCI	6 months	High-intensity resistance	↑ Global cognition and certain aspects of executive cognition
The SYNERGIC Study [82,84,104]	175, 120, 175	MCI	20 weeks	Aerobic, resistance + cognitive training	↑ Global cognition, e.g., gait performance; functional brain connectivity
[85]	155	MCI	12 months	Resistance	↑ Cognitive executive function (Stroop test)
[88]	555	MCI	12 months	Structured lifestyle (physical) activity	↑ Global cognition and memory
[83]	308	MCI	12 months	Multicomponent	↑ Global cognition and MMSE, ↓ temporal lobe atrophy
[77]	323	MCI	Daily physical actiivity	Moderate–high aerobic	↓ Brain atrophy
**C. Mild–Moderate Alzheimer’s Disease**					
**Study**	**N**	**Population/Stage**	**Duration**	**Exercise Type**	**Key Outcomes**
ADEX [91]	200	Mild AD	16 weeks	Moderate-to-high-intensity aerobic	↓ NPI, possible cognitive benefit with high intensity exercise and adherence
FIT-AD [92,105]	90	Mild–moderate AD	6 months	Moderate–high aerobic cycling	↓ White matter hyperintensity progression (not the brain volume). No cognitive benefit, large inter-individual differences
DAPA [96,97,98]	494	Mild–moderate AD	12 months	Moderate–hard aerobic and resistance	No cognitive benefit, slight worsening of cognition, and reduction in QALY
[99,100]	210	AD	12 months	Home multicomponent	Slow decline (functional independence and executive function), no other cognitive benefit
[95]	72	AD	12-week	Multicomponent	↑ Cognition and ↓ depression

Note: AD: Alzheimer’s disease; global cognition is measured by ADAS-Cog-13 (Alzheimer’s disease assessment-cognitive subscales-13 item version). QALY: quality-adjusted life year; multicomponent exercise: aerobic, resistance and balance and executive functioning training; MET: memory enhancement training; MMSE: mini-mental state examination; NPI: neuropsychiatric inventory; CVD: cardiovascular disease; VEGF: vascular epithelial growth factor; ↑, increase; ↓, decrease.

### 2.3. Timing of Exercise Intervention Is Critical for Prevention or Delay of Progression in AD

The timing of exercise initiation, along with physiological responsiveness to the intervention, emerges as a key determinant of outcomes in human studies (Table 2). Converging evidence indicates that interventions delivered earlier—particularly during preclinical AD or mild cognitive impairment (MCI)—yield more substantial and durable cognitive benefits than those implemented in later stages, where extensive synaptic and neuronal loss limits reversibility, such as mild-to-moderate or advanced dementia [16,91]. This pattern supports the principle that exercise is more effective at promoting brain health and cognitive reserve before extensive synaptic and neuronal loss occurs, rather than attempting to reverse well-established neurodegeneration. For instance, in the ADEX trial, a 16-week moderate-to-high intensity aerobic exercise program in individuals with mild AD produced physiological benefits, including improved cardiorespiratory fitness and functional ability [106]. However, no significant overall improvements were observed in global cognition or executive function [107]. Furthermore, cognitive benefits appear more limited in moderate dementia compared to milder stages or preclinical phases. Studies in more advanced dementia often demonstrate preserved or improved functional/motor outcomes (e.g., gait, activities of daily living) but minimal to no significant effects on global cognition or executive domains, even with high-intensity protocols [108]. This differential pattern reinforces the hypothesis that while functional benefits may persist into later disease stages, robust cognitive enhancements depend on earlier intervention when neural reserve remains relatively intact; this is confirmed in mouse models of AD studies, as for example AD mice that started aerobic exercise at 2 months old showed significantly improved glymphatic clearance of extracellular Aβ toxic proteins, but not when the intervention started at 7 months old [109].

Exercise form and intensity may further modulate outcomes. Aerobic exercise and resistance exercise showed the greatest benefits on global cognition and executive function respectively, whereas mind–body exercise benefitted memory [110]. However, feasibility, safety, and tolerability concerns limit high-intensity exercise in older adults with established dementia or comorbidities, leaving the generalizability of intensity effects uncertain in clinical AD populations [74].

Taken together, these findings support positioning exercise primarily as a preventive or early-stage strategy to delay onset and slow progression, rather than a reversal intervention in established dementia. This perspective aligns with models of cognitive reserve and multidomain lifestyle prevention, where sustained physical and cognitive engagement exerts additive or synergistic protective effects on neural resilience across the lifespan. Future trials should prioritize early recruitment (preclinical/MCI) and personalized dosing to maximize neuroprotective potential.

#### The Critical Window of Exercise Intervention for AD Prevention in Middle-Aged Adults

Neurodegenerative processes associated with AD are estimated to begin approximately two decades before clinical symptoms emerge, positioning midlife (∼40–65) as a critical window for intervention [5,11]. This period is characterized by a high prevalence of modifiable risk factors, including physical inactivity, obesity, hypertension, diabetes, and adverse lifestyle behaviors such as smoking. Physical activity is consistently identified as a protective factor, improving cognitive function and reducing the risk of AD in a dose-dependent manner through beneficial effects on cardiometabolic health across the aging process, even at low frequencies (e.g., a few times per month), when initiated in midlife and beyond [11,111,112]. Aerobic exercise reduces aortic characteristic impedance, improving glucose homeostasis, blood pressure, and cardiovascular fitness in middle-aged adults (30–64 years old), all of which are linked to cognitive outcomes partly via increased brain-derived neurotrophic factor (BDNF), a key mediator of synaptic function and memory, while also preserving skeletal muscle mass, which declines during midlife [113,114,115]. Combined aerobic and resistance exercise enhances cognitive performance and metabolic regulation, with strong effects in type 2 diabetes, sedentary and overweight populations [116,117].

A structural study with MRI in a RCT in healthy adults (50–70, average age 58) has shown that multimodal (computerized) cognitive training enhances brain volume in the pecuneus, a cognitive area of the medial parietal hub of the default mode network (DMN), adjacent to the posterior cingulate cortex [118]. Using functional MRI, multimodal interventions combining physical and cognitive training for the same cohort appear to yield additive neuroprotective effects at resting state, change the functional connectivity mostly by increasing connections among frontal, temporal and parietal lobes within large-scale brain networks such as the DMN and supporting their potential utility in mitigating early neurodegenerative changes during late midlife [119]. In addition, higher physical activity levels are associated with reduced concentrations of AD-related amyloid-β burden in cognitively normal populations, in particular older women, and Tau protein levels in middle-aged people with or without MCI [69,120,121].

## 3. Forms of Exercise and Their Links to Cognitive Benefits

Growing evidence indicates that different exercise modalities confer distinct yet overlapping cognitive benefits in aging and AD, varying by degree of physical exertion, cognitive engagement, and emotional/attentional components. These differences suggest partially shared (e.g., neurotrophic upregulation, anti-inflammatory effects) but modality-specific mechanisms (e.g., mind–body for emotion regulation, dual-task for executive coordination). This section reviews and compares aerobic, resistance, dual-task, and multimodal (or multidomain or muti-component) exercises and mind–body exercises, evaluating their contributions to cognitive function in individuals diagnosed with AD.

### 3.1. Aerobic and Resistance Exercise and Their Benefits

Aerobic exercise entails rhythmic, continuous movements of large muscle groups of one’s body to improve mood-alleviating NPS, and resistance exercise applies external loads to induce skeletal muscle hypertrophy and strength gains, associated with increased cortical volume, neuroplasticity and cognition [79]. Mechanistically, aerobic exercise is primarily linked to hippocampal gene regulation and peripheral BDNF upregulation and reductions in TNF-α and IL-15 levels. Yet, both aerobic and resistance exercise are consistently associated with enhanced neuroplasticity substrates (neurogenesis, neurotrophic signaling, inflammation, antioxidant defense and stress response), i.e., hippocampal plasticity and neuronal regeneration via stimulating expression of trophic factors like BDNF, IGF-1 and neurotransmitters like dopamine, executive function via muscle contraction (myokines), reduced neuroinflammation and oxidative stress (anti-inflammatory cytokines IL-10 and TGF-β), and improved cerebral and vascular repair through systematic metabolic and neuroimmune homeostasis in aging and neurodegenerative contexts (see Figure 1) [13,122,123,124,125]. In addition, physical exercise improves mood (reducing stress and anxiety), sleep, and insulin resistance leading to improved overall health and brain functions [123,124,125]. In mild AD, moderate-to-high intensity aerobic programs attenuate hippocampal atrophy rates and support functional gains [80]. Meta-analyses of RCTs in cognitive-normal older adults and those with AD indicate that aerobic exercise improves global cognition, particularly memory function (e.g., MMSE gains), with optimal dosing of ~ 30 min per session ≤ 150 min/week, in up to three sessions per week, or 60 min per session up to twice per week [126,127].

Resistance exercise complements aerobic exercise in its unique signal transduction pathway (e.g., increased Akt signaling) by overall enhancing cognitive and inhibitory control and is recommended for the elderly population who is at risk for AD [5] to improve their muscle strength and balance, functional autonomy and quality of life [46,60,79,124,125,127,128]. Resistance exercise reduces the risk for developing MCI and AD by increasing neurotrophic factors, insulin sensitivity and neurogenesis, and in the meantime it reduces neuroinflammation, toxic Aβ load and neurofibrillary tangles [129,130]. For example, in MCI, high-intensity resistance exercise (2–3 days/week for 6 months) significantly improved global cognitive and executive function in a SMART clinical trial [81]. Further, a combination of physical and mental exercises offers the greatest benefits in improving working memory and task-switching ability [127].

### 3.2. Mind–Body Exercises and Their Benefits

Mind–body practices such as Tai Chi, mindful Tai Chi Chuan (MTCC), Baduanjin and yoga integrate physical movement, breath control, mindfulness, and often social elements, yielding multimodal benefits in global cognition and executive function [120,131]. Among these practices, MTCC outperformed traditional Tai Chi or standalone mindfulness in enhancing global cognition and reducing cognitive frailty in older adults at risk for AD [22]. A review with meta-analysis of 14 RCTs showed that Baduanjin exercise (a traditional Chinese mind–body exercise) improved global cognitive function, memory, processing speed and executive function [131]. Another review with meta-analysis of 2565 cases including different types of mind–body exercises (Tai Chi, dance, qigong) for a duration of 8–36 weeks showed significantly improved cognitive scores (MoCA and MMSE and trail making test-A and -B scores), depressive status and balance, with reduced CSF Tau protein levels in middle-aged and older adults with MCI [120]. At the brain circuitry level, yoga practice increased anterior hippocampal connectivity within memory and emotion regulation networks [101]. Recent imaging data also suggest that interactive, exergame-based “Brain-IT” training with biofeedback breathing positively induced structural brain changes in both gray and white matter integrity in the hippocampus, thalamus and anterior cingulate cortex in correlation with the cognitive performance [86]. The multisensory and integrated nature of these interventions likely concurrently modulates neurotrophic factors, vascular health, and inflammation—key protective pathways in AD—explaining broad psychosocial and cognitive advantages.

### 3.3. Dual-Task and Multimodal Exercise and Their Benefits

Dual-task paradigms pair physical activity (e.g., walking) with concurrent cognitive demands (e.g., attention/memory tasks), engaging overlapping motor–cognitive networks with synergistic effects. Multimodal interventions integrating cognitive challenges with movement enhance functional connectivity, like frontal–hippocampal connectivity, more than single-modality exercise [82,83,84,104,132]. Specifically, complex mental activities have shown to be associated with reduced hippocampal atrophy and preserved cognitive reserve, whereas regular aerobic physical activities are associated with reduced cortical brain volume loss in aging, including in MCI and early-stage AD [133]. These paradigms synergistically stimulate attention, working or episodic memory, and emotional regulation through coordinated neural activation, positioning them as promising early-stage strategies. A higher frequency of over five sessions per week may lead to greater cognitive improvements based on meta-regression and meta-analysis of 23 clinical trials [134].

Among the paradigm of multimodal exercise, square dance, which is popular in middle-aged and older Chinese women, stands out as a recommendation for the prevention of MCI, dementia and associated depressive symptoms, as it exemplifies a multimodal intervention combining moderate-intensity aerobic activity, rhythmic coordination, balance demands, and strong social/cognitive engagement. The social interaction promotes cognitive improvement through enhanced executive function, hippocampal plasticity, and psychosocial stress attenuation. In cognitively healthy middle-aged and older Chinese women, long-term (multi-year) participation preserves mental cognitive capacity [103,135]. Data from China have shown that leisure-time physical activity dominated by square dancing correlates positively with performance across all MMSE domains and with lower MCI prevalence, similar to the effect of the Tai Chi group (see Section 3.2) [136,137]. Systematic reviews and meta-analyses support these benefits. A synthesis of 24 (primarily non-English) studies indicates improvements in physical, mental, and cognitive function, with recommendations for mixed-gender and intergenerational practice [102]. A 2025 network meta-analysis of 28 studies in adults > 60 years found square dancing particularly effective for mental health outcomes, while ballroom and square dance ranked highly for cognitive reserve [103].

Randomized evidence further strengthens its utility in older populations. A single-blinded RCT demonstrated that square dancing (2×/week for 12 weeks) improved cardiometabolic markers (↓BMI, LDL, fat mass; ↑basal metabolic rate) and physical performance (SPPB) and produced modest cognitive gains, potentially via the muscle–brain axis [138]. In MCI cohorts, 3-month programs enhanced overall cognition (measured by MoCA-Peking version, including attention language, verbal memory, visuospatial function, executive function and orientation) and reduced depressive symptoms [103]. Pilot studies confirm high acceptability and feasibility for community-dwelling older adults with MCI and depression [139,140]. An imaging study showed that 3-month Chinese group dancing interventions increased hippocampal volume and episodic memory in amnestic MCI [141].

These findings emphasize mind–body and dual-task modalities (e.g., Tai Chi, yoga) and support early, accessible, group-based exercise prescriptions. Square dancing’s low cost, scalability, and cultural acceptance make it a promising neuroprotective strategy, particularly in Asian populations.

### 3.4. Comparative Effectiveness Across Modalities

Large-scale syntheses highlight modality-specific profiles. In AD and MCI, multicomponent, dual-task, and cognitively enriched programs frequently outperform single-modality aerobic or resistance training for global cognition and executive domains, though effect sizes remain modest and heterogeneous [16,133]. Mind–body modalities excel in emotion regulation, frailty reduction, and hippocampal network connectivity, while aerobic/resistance emphasize structural and metabolic protection [82,83,84,91,104,122,132]. Adjunctive strategies (e.g., diet) may further enhance outcomes [67]. In fact, current clinical trials are targeting multidomain treatment to prevent or delay the progression of AD, such as the AU-ARROW study which follows the Finnish (FINGER) RCT model [142].

Collectively, modality, intensity, cognitive engagement, and timing critically determine cognitive benefits in aging and AD (see Figure 1 for mechanistic convergence). These insights underscore the value of personalized prescriptions, with multicomponent/dual-task often optimal for early intervention. The biological underpinnings of these modality differences are explored next.

## 4. Mechanisms of the Neuroprotective Role of Exercise

### 4.1. Changes in Structural and Functional Brain Integrity Induced by Exercise in Humans

Brain white matter integrity is critical for cognition and disconnection or degeneration of these white matter tracts is observed in aging AD, particularly in pathways such as the uncinate fasciculus and cingulum bundle that connect frontal, temporal, and parietal regions [143,144]. Physical exercise, particularly aerobic and mind–body modalities, is associated with preservation of brain structure—especially white matter volume and integrity (e.g., thalamic radiation and corpus callosum)—as well as functional integrity and episodic memory, including in older adults at risk for MCI or in the early stages of AD [64,65,86,145]. In healthy older adults, aerobic walking and dance training are linked to better preservation of white matter integrity in the frontal and temporal lobes and improved short-term memory, in tracts vulnerable to age-related degeneration [144]. In cross-sectional analyses, individuals with greater cardiorespiratory fitness exhibit less age-related decline in white matter microstructure across widespread brain regions. Intervention data further suggest that 6 months of aerobic exercise or progressive high-intensity resistance exercise may increase the brain volume in areas for memory, such as the hippocampus, in individuals with (probable) MCI, but not for older adults with mild–moderate AD (Table 2A) [64,69,80,146,147]. Functional imaging provides complementary evidence of exercise-induced reorganization of neural networks. For example, Kundalini yoga and cognitive memory interventions have been shown to enhance hippocampal connectivity within the ventral visual stream, the DMN and frontoparietal networks supporting memory and emotion regulation in older women at risk for AD [101], suggesting benefits for both cognitive and affective processing in aging. The 3-month aerobic dance program increased hippocampus–hub temporal network connectivity within DMN in elderly people with aMCI [148].

Molecular and biomarker studies reinforce the theory that these structural and functional effects are mediated through enhanced neurotrophic support, reduced neuroinflammation, and improved network organization by showing exercise-related enhancements in neuroplasticity (Table 3). Aerobic training is associated with favorable changes in peripheral markers of neurotrophic signaling (e.g., BDNF pathways), myokine cathepsin B and synaptic maintenance proteins in neuron-derived extracellular vesicles [20,149]. These adaptations align with preserved neuronal health and reduced inflammatory signatures.

Emerging evidence points to systemic mechanisms, including modulation of the gut–brain axis through the Nrf2 signal pathway [150]. Exercise alters gut microbiota composition in ways that correlate with enhanced neurotrophic signaling and hippocampal structural integrity, providing a peripheral-to-central pathway for neuroprotection [20].

**Table 3:** Multi-level mechanisms underlying the neuroprotective effects of exercise.

Level	Mechanism	Key Mediators/ Pathways	Main Effects in AD Context	Supporting Evidence (Selected References)	Stage-Specific Notes
Molecular/Cellular	Neurotrophic signaling	BDNF-TrkB, CREB-BDNF, proBDNF	↑ Synaptic plasticity, neurogenesis, dendritic spine density; memory preservation	[3,12,124]	Most robust in preclinical/MCI; ↓ sensitivity in advanced AD
Molecular/Cellular	Anti-inflammatory effects	↓ TNF-α, IL-6, NF-κB; ↓ microglial/astrocyte activation; ↑ IL-10	↓ ER stress, downstream inflammatory signaling, ↓ neuronal damage, shifted to neuroprotective milieu	[23,35,56,124,151]	HIIT favors neuroprotective astrocyte phenotype
Cellular/Bioenergetic	Mitochondrial biogenesis and iron homeostasis	PGC-1α/NRF-1 (biogenesis); mitophagy; ↑ antioxidant defenses	↑ Mitochondrial density & efficiency; ↓ ROS/oxidative stress; preserved ATP production	[32,33,34,124,152]	Dual biogenesis + mitophagy counters AD mitochondrial fragmentation/damage
Cellular	Autophagy	Irisin-mediated autophagy-activating pathways including AMPK	↑ Neuroplasticity	[36,153]	↑ Autophagy in the hippocampus alleviating AD pathology
Systemic/Structural	Vascular & network preservation	↑ Cerebral blood flow; preserved hippocampal/white matter integrity	Enhanced perfusion, network coherence (esp. mind–body modalities), ↓central arterial stiffness	[101,154,155]	Stabilizes early-stage atrophy; mind–body enhances emotion/memory networks
Integrated Outcome	Multi-target convergence	Overlapping pathways (neurogenesis, etc., see Figure 1)	↑ Neurogenesis and neuronal resilience, cognitive reserve; slowed progression (esp. early intervention)	[15,16,40,63,69]	Greatest efficacy in preclinical/MCI stages; more modest effects in moderate–advanced AD stage
Systemic/Integrative	Exerkine-mediated signaling	↑ Exerkines, e.g., myokines (those derived from muscle)	Peripheral-to-central communication: ↑ neuronal survival, ↓ inflammation, ↑ plasticity	[20,38,63,113,149,156,157]	↑Neuroprotective proteins in NDEVs (BDNF, humanin); *APOE* ε4 carriers show amplified response
Integrative/Organ level	Gut microbiota	Gut–brain axis through metabolic, immune, neural, and endocrine pathways and Nrf2 signaling	↑ Cellular antioxidant defense, mitochondrial function and anti-inflammation	[17,29,150,158]	Diet and exercise
Integrative/Organ system level	Meningeal lymphatic vessel and glymphatic flow	AQP4-mediated glymphatic flow	↓ Extracellular Aβ toxic protein Converting to neuroprotective astrocyte type polarization of both astrocytes and microglia, enhances their phagocytic abilities	[39,41,109,159,160]	Most robust in early stage/MCI; compromised in advanced AD
Metabolic	Reducing glucose level	Increasing insulin sensitivity	↑ IGF-1 and cognition ↓ GSK-3β	[26]	Diet and exercise

Note: BDNF, brain-derived neurotrophic factor; TrkB, tropomyosin receptor kinase B; MCI, mild cognitive impairment; AD, Alzheimer’s disease; TNF-α, tumor necrosis factor-α; IL, interleukin; NF-κB, nuclear factor kappa-light-chain-enhancer of activated B cells; ER, endoplasmic reticulum; HIIT, high-intensity interval training; PGC-1α, peroxisome proliferator-activated receptor gamma coactivator-1 alpha; NRF-1, nuclear respirator factor 1; ROS, reactive oxygen species; AMPK, adenosine monophosphate-activated protein kinase; NDEV, neuron-derived extracellular vesicle; *APOE* ε4, apolipoprotein E gene epsilon 4 variant; AQP4, aquaporin-4 water channel; IGF, insulin-like growth factor; GSK-3β, glycogen synthase kinase-3beta; Nrf2, nuclear factor erythroid 2-related factor 2.

Collectively, these findings indicate that regular physical exercise supports brain integrity through multiple converging mechanisms, such as preservation of white matter microstructure, enhanced functional network coherence, upregulated neurotrophic processes, and gut-mediated influences, and they are associated with enhancements in key cognitive domains, including episodic memory, executive function, attention, and mood/emotion regulation, particularly in older adults at risk for AD or in preclinical/mild cognitive impairment (MCI) stages [8,22,88,101,147]. Such effects position exercise as a promising strategy for maintaining brain resilience against age- and AD-related degeneration (Figure 1).

### 4.2. Multi-Level Mechanisms of the Neuroprotective Role of Exercise

Molecular evidence links the functional gains to exercise-induced neuroprotection. In the ADEX randomized controlled trial of moderate-to-high intensity aerobic exercise in individuals with mild-to-moderate AD, neuron-derived extracellular vesicles (NDEVs) isolated from plasma showed significant post-intervention increases in neuroprotective proteins (proBDNF, BDNF, and humanin), with particularly robust responses in *APOE* ε4 carriers [20]. These changes suggest that exercise promotes peripheral-to-central signaling that upregulates neurotrophic support, enhances synaptic resilience, and mitigates inflammatory processes within neuronal compartments.

Broader mechanistic reviews delineate exercise effects across multiple pathophysiological domains implicated in AD [15,122,124] (also see Section 1.1). These include intercellular signaling and synaptic plasticity, immune and inflammatory responses, metabolic and oxidative stress pathways and mitochondrial bioenergetics, endothelial and cerebrovascular function, apoptosis (programmed cell death), DNA damage response and repair pathways, and cytoskeletal and membrane protein regulation (see Figure 1 for an integrated schematic of these converging mechanisms). By modulating this heterogeneous set of pathways, exercise offers a multi-target approach to counteract neurodegeneration and bolster neuronal resilience, synaptic integrity, and cognitive reserve with the potential to prevent onset, stabilize function, or slow progression in early disease stages, and has the greatest efficacy in preclinical/MCI stages (Figure 1 and Table 3).

Regular physical exercise (e.g., aerobic and resistance training) induces the release of peripheral factors, including exerkines (e.g., irisin, myokines, IL-6) and metabolic signals, which communicate with the brain via peripheral-to-central signaling pathways. These signals act through multiple metabolic axes (muscle–brain, gut–brain, liver–brain, and spleen–brain) to enhance key neuroprotective mechanisms, including increased autophagy via inhibition of phosphoinositol-3-kinase (PI3k)/protein kinase B (Akt)/mechanistic target of rapamycin (mTOR) pathway and activation of nuclear factor eythroid-2-related factor 2 (Nrf2) signaling pathways to remove damaged proteins and organelles, upregulate antioxidant and detoxifying genes, and improve blood–brain barrier (BBB) integrity. Downstream adaptations fall into several interconnected processes: (1) enhanced neurotrophic signaling, e.g., brain-derived neurotrophic factor (BDNF)–tropomyosin receptor kinase B (TrkB) signaling, neurogenesis, and synaptic plasticity; (2) improved mitochondrial and oxidative regulation via activation of AMP-activated protein kinase (AMPK)/peroxisome proliferator-activated receptor gamma coactivator −1 alpha (PGC-1α)/nuclear respiratory factor 1 (NRF-1)-mediated mitochondrial biogenesis, mitophagy, and reduced reactive oxygen species (ROS); (3) increased glymphatic and meningeal vessel function; (4) restoration of metabolic homeostasis (reduced insulin resistance glycogen synthase kinase 3-beta (GSK-3β) activity), and increased IGF-1 signaling; (5) modulation of gut microbiota (increased microbial diversity, short-chain-fatty acid (SCFA) production with reduced neuroinflammation). Collectively, these effects suppress neuroinflammatory pathways (e.g., decreased pro-inflammatory cytokines such as TNF-α and IL-6, nuclear factor kappa-light-chain-enhancer of activated B cells (NF-κB) pathway activity, and microglial activation) and enhance amyloid-β removal, leading to improved neuronal survival. Systemically, these exercise-mediated changes culminate in improved cerebral blood flow and vascular function, preserved hippocampal volume and enhanced white matter structural integrity and stress regulation, and translate into cognitive benefits, increased cognitive reserve especially executive function and memory and a reduced rate of cognitive decline preventing, delaying or slowing AD progression when implemented early in the disease continuum (Table 3).

#### 4.2.1. Neurotrophic Factors and Synaptic Plasticity in Link to Exercise

BDNF upregulation is a central linking exercise to enhanced synaptic plasticity, neurogenesis, and cognitive preservation. Exercise activates BDNF-TrkB signaling, promoting dendritic spine density, synaptic strengthening, and hippocampal neurogenesis—processes disrupted in AD [12]. In human and animal models, elevated BDNF correlates with improved cognition, learning and memory [56,149,161]. However, BDNF pathway sensitivity declines as AD pathology advances, contributing to diminished exercise efficacy in later stages [124]. Aerobic exercise stimulates intracellular cascades (e.g., CREB-BDNF and PGC-1α pathways) that couple metabolic regulation to synaptic function [162]. These molecular adaptations align with neuroimaging evidence of preserved or modestly increased hippocampal volume post-exercise, supporting long-term memory benefits in early/preclinical stages.

#### 4.2.2. Reduction in Neuroinflammation in Link to Exercise

Chronic neuroinflammation drives AD progression, with elevated pro-inflammatory cytokines (TNF-α, IL-6) and NF-κB pathway activation promoting microglial/astrocyte reactivity and neuronal damage. Exercise attenuates this cascade, shifting toward an anti-inflammatory, neuroprotective milieu. In AD mouse models, physical activity reduces hippocampal inflammatory signaling and improves spatial memory [56]. Human studies confirm that yoga lowers inflammatory gene expression in older women at risk for AD [23]. Aerobic and resistance modalities decrease TNF-α, IL-1β, and IL-6 while elevating anti-inflammatory IL-10 [42,124]. High-intensity interval training in AD rats and mice reduces overactivation of glial cells, prevents neurotoxic, pro-inflammatory M1 astrocyte polarization and favors anti-inflammatory, neuroprotective M2 phenotypes, enhancing synaptic maintenance and clearance of toxic Aβ and p-tau proteins through the glymphatic system and the kidney [151]. These effects extend beyond cytokine suppression to improved mitochondrial redox balance and exerkine-mediated signaling, collectively reorganizing neuroimmune dynamics toward neuronal survival [63,124].

#### 4.2.3. Mitochondrial Quality Control and Bioenergetics in Link to Exercise

Mitochondrial dysfunction—characterized by mitochondrial disturbances (reduced ATP and Ca^2+^ handling, increased reactive oxygen species ROS) and altered mitochondrial axonal transport depriving mitochondria from presynaptic terminals by accumulated damage from Aβ and pathogenic tau, with reduced size and fragmented cristae, leading to impaired neurotransmitter release —is a core AD hallmark [163]. Exercise counters this via enhanced mitochondrial quality control. In AD models, physical activity promotes biogenesis through PGC-1α/NRF-1 activation, increases mitochondrial density, boosts ATP efficiency, and stimulates mitophagy to clear dysfunctional organelles [152]. These actions reduce ROS, mitigate oxidative damage, and sustain neuronal metabolic homeostasis amid neurodegenerative stress [124]. By preserving mitochondrial integrity, exercise supports synaptic function and long-term neuronal viability, particularly when initiated early.

#### 4.2.4. Exerkines as Systemic Mediators in Exercise

Exerkines, bioactive molecules (e.g.,myokines) released from the skeletal muscle and peripheral tissues such as liver during exercise, bridge peripheral adaptations to central neuroprotection [28,30,42,113,157]. Aerobic exercise encourages the release of myokine cathepsin B (CTSB) in late middle-aged adults at genetic risk of Alzheimer’s disease [149]. Aerobic and resistance training elevate circulating exerkines, promoting neuronal survival, reducing neuroinflammation, and modulating synaptic plasticity [63]. While much exerkine research focuses on Parkinson’s disease, overlapping pathways (reduced protein aggregation, improved metabolic resilience, enhanced neuroplasticity) apply to AD. Exerkines integrate neurotrophic, inflammatory, and mitochondrial signals, explaining systemic-to-CNS benefits [124]. Supporting this, structured exercise in mild–moderate AD increases neuroprotective proteins (proBDNF, BDNF, humanin) in neuron-derived extracellular vesicles (NDEVs), with robust responses in *APOE* ε4 carriers, alongside reduced inflammatory signatures, which implicates exerkine-mediated signaling in molecular and cognitive gains in human trials [20,42,156]. A mouse study also showed that exercise triggers skeletal muscle-derived extracellular vesicles secretion, which are beneficial for the clearance of amyloid plaques and preservation of cognitive function [156].

#### 4.2.5. Exercise Modulation of Gut–Brain Axis and Brain Waste Clearance Systems (Meningeal Lymphatics and Glymphatic System)

Emerging evidence indicates that exercise exerts neuroprotective effects, ameliorating the reduction in hippocampal neurogenesis in AD partly through modulation of the microbiota–gut–brain axis (MGBA) in murine models and enhancement of brain waste clearance via the meningeal lymphatic and glymphatic systems [164,165]. Gut microbiota activate the nuclear factor erythroid 2-related factor 2 (Nrf2) signaling pathway, a key regulator of mitochondrial function, and anti-inflammatory response processes that are impaired in AD. Exercise stimulates this gut microbiota-mediated transformation of dietary and host-derived substrates into bioactive metabolites, which may counteract neurodegeneration through activation of the Nrf2 pathway [150]. As we get older, our microbiome goes through compositional changes with reduced microbial diversity, for example, increased Protecbacteria and decreased Firmicutes, particularly in menopausal women [166]. In both rodent AD models and human observational studies, structured aerobic and resistance exercise increase microbial diversity and alter microbiota composition, promote short-chain fatty acid (SCFA)-producing taxa (e.g., Akkermansia muciniphila, Faecalibacterium), elevate short-chain fatty acid levels, strengthen intestinal barrier integrity, and reduce systemic neuroinflammation through the influence of miRNA expression, thereby supporting BBB function and hippocampal neuroplasticity [158,165,167].

Concurrently, long-term exercise enhances meningeal lymphatic and glymphatic (glia-mediated cerebral lymphatic vessels) drainage capacity to clear toxic proteins including β-amyloid [41,159,160,168,169]. Based on the glymphatic hypothesis, the water channel protein aquaporin 4 (AQP4), located at the end feet of the astrocyte membrane, facilitates the clearance of interstitial solutes including β-amyloid from the brain. However, in the mid or late stage of AD, glymphatic function is impaired due to loss of AQP4 polarization, disruption of structural and functional integrity of the system and shortened sleep architecture which further aggravate this disruption. In the APP/PS1 mouse model and STZ-treated rat model of AD, swimming or wheel aerobic exercise and high-intensity treadmill training can improve learning and memory by improving the hippocampal AQP4 polarization-mediated system to clear Aβ deposition, and this polarization is regulated by neuroprotective astrocyte phenotypes [109,151,170]. This improvement was most pronounced when aerobic exercise intervention was initiated in younger mice, with greater efficacy at 3 months compared to 7 months of age at the onset of training [109]. Human imaging studies using contrast-enhanced MRI further demonstrate that regular aerobic training augments putative glymphatic influx and meningeal lymphatic flow, correlating with better cognitive outcomes. These effects are mechanistically linked to improved AQP4 polarization on astrocytic end feet, increased perivascular CSF-ISF exchange, and synergistic interactions with neurotrophic (BDNF) and anti-inflammatory pathways already discussed [171]. Collectively, these peripheral-to-central mechanisms position exercise as a multi-target intervention that not only upregulates BDNF and mitochondrial biogenesis but also optimizes waste clearance and gut-derived metabolite signaling, potentially slowing AD progression when initiated early.

### 4.3. Gender Differences in the Neuroprotective Role of Exercise

Women comprise approximately two-thirds of all Alzheimer’s disease (AD) cases worldwide, reflecting a lifetime risk after age 65 that is nearly twice as high as in men (see review [125]). This disparity is not fully explained by longevity alone; it arises from a complex interplay of biological and psychosocial factors. Steroid hormones play a central role: estrogen exerts robust neuroprotective effects in women through enhancement of synaptic plasticity, neuronal survival, cardiovascular integrity, and regulation of amyloid-β clearance and tau phosphorylation which may be mediated through FNDC5/irisin and BDNF signaling pathways [172]. In contrast to the gradual age-related decline in testosterone observed in men, women experience a precipitous reduction in estrogen during perimenopause and menopause, typically in midlife. This hormone shift accelerates hippocampal atrophy, disrupts calcium homeostasis, and promotes faster progression of AD neuropathology, with verbal and working memory being particularly affected [173]. Exercise, especially strength training and multicomponent programs, has been shown to exert favorable effects on global cognition in older women.

Genetic interactions amplify this vulnerability. Female *APOE* ε4 carriers exhibit heightened risk, with more rapid neurofibrillary tangle (NFT) progression and elevated plasma tauopathy at equivalent amyloid burdens (referred to as A^+^T^+^) compared with male carriers. This manifests as greater hippocampal atrophy, cortical thinning, and metabolic abnormalities in older women [174]. Women also face disproportionate psychosocial stressors: as primary caregivers for family members with dementia, they experience higher levels of chronic stress, sleep disruption, and neuropsychiatric symptoms (NPS), all of which independently elevate AD risk and exacerbate disease burden [125].

These sex-specific vulnerabilities translate into differential responsiveness to exercise interventions. Women over 60 years of age consistently derive greater cognitive benefits—particularly in executive function, visuospatial abilities, and emotional regulation—across aerobic, resistance, and multimodal training modalities. Resistance exercise elicits more pronounced gains in grip strength, endurance, and functional capacity in women, indicating higher physiological adaptability. Women also engage more frequently in light-intensity activities such as daily walking, which is associated with increased hippocampal volume in females but not males. Moderate-intensity aerobic training preferentially improves executive function in women, whereas benefits in men are more aligned with gains in general strength, cardiopulmonary fitness, and metabolic regulation. However, men benefit more from computerized cognitive training than women in changing brain volume [118]. Overall, exercise appears to confer more pronounced neuroprotective effects on cognitive function, emotional health, and neural structure in women, likely via sex steroid-dependent modulation of BDNF/irisin signaling, anti-inflammatory pathways, and mitochondrial resilience [125,172].

## 5. Discussion and Conclusions

The evidence synthesized across human clinical trials, observational studies, and animal models consistently supports exercise as a multifaceted, non-pharmacological intervention capable of delaying AD onset, slowing progression, and preserving cognitive function, particularly when initiated early. Timing emerges as a critical determinant: interventions during preclinical stages or MCI, when cognitive reserve and neural integrity remain relatively intact, yield the greatest magnitude of cognitive, structural, and functional benefits [16] (Table 2). In contrast, exercise implemented in well-established AD more often stabilizes physical performance, activities of daily living, and neuropsychiatric symptoms, while producing more limited or domain-specific cognitive gains [91]. However, translational challenges persist, including differences in age and comorbidity burden: most animal models use young rodents with minimal comorbidities, whereas human participants are typically older with extensive pathology. Additional barriers include protocol heterogeneity (intensity, frequency, duration, modality), small sample sizes, inconsistent outcome measures, variable adherence, and potential sex-related differences in response—all contributing to trial discrepancies.

Mind–body (e.g., Tai Chi, yoga) and technology-assisted modalities offer integrated physical, cognitive, and emotional benefits, enhancing hippocampal connectivity, emotion regulation, and frailty reduction [22,86,101]. Multicomponent/dual-task approaches often outperform single-modality training for executive function and global cognition in MCI/early AD [16]. Optimal prescriptions involve varied intensity and multiple weekly sessions, ideally starting in preclinical/MCI phases.

Underlying mechanisms converge on neurotrophic upregulation (e.g., BDNF signaling), reduced neuroinflammation, enhanced mitochondrial quality control (biogenesis/mitophagy), preserved synaptic integrity, and exerkine-mediated peripheral-to-central communication (see Figure 1 [63,124,152]). These mechanisms translate into increased physical fitness, neuroplasticity, and preservation of hippocampal, cortical thickness, and white matter structure/function in human trials [69,91]. Recent umbrella reviews and meta-analyses provide compelling cumulative evidence that exercise positively influences AD prevention and management, including reduced dementia risk [16]. Exercise thus represents an accessible, low-cost, multi-target strategy for promoting brain health across the lifespan, with particular promise in at-risk individuals.

In conclusion, while not curative, regular physical exercise—especially early, multimodal, and sustained—offers robust neuroprotective potential to delay AD progression, preserve cognitive reserve, and enhance quality of life.

## 6. Limitations and Future Directions

Despite substantial evidence supporting exercise as a neuroprotective strategy in Alzheimer’s disease (AD), several methodological and practical limitations constrain its translation into routine clinical practice and standardized guidelines.

Many RCTs suffer from small sample sizes, reducing statistical power and generalizability, particularly for moderate-to-advanced dementia where recruitment and retention are challenging [92,108]. Heterogeneity in exercise “dosing” (intensity, frequency, session duration, modality) complicates direct comparisons and hinders the development of universal prescriptions [16,91]. Variability in cognitive/functional outcome measures further impedes meta-synthesis [88,101]. Study populations often lack diversity in race/ethnicity, socioeconomic status, and comorbidities, limiting external validity [16]. Multimodal interventions (e.g., yoga, Tai Chi, dual-task) are frequently single-site with homogeneous samples, restricting broader applicability [22,101].

Adherence poses a major challenge in AD, often biasing results toward healthier/more motivated participants and underestimating real-world effects [88,108]. Mechanistic studies rely heavily on short-term interventions, peripheral blood biomarkers, or animal models, raising questions about long-term human translation [2,18,152].

Despite these constraints, exercise holds tremendous potential as a core lifestyle recommendation in AD management. Future research should prioritize the following:

1. Large-scale, multicenter, longitudinal RCTs with diverse, representative populations and extended follow-up to clarify long-term effects on progression, mechanisms, and functional outcomes;
2. Standardized protocols using wearable technologies for objective dosing/adherence monitoring;
3. Personalized prescriptions tailored to disease stage, genotype (e.g., *APOE*), comorbidities, and preferences;
4. Integration of exercise with cognitive, dietary, or other multidomain interventions to maximize synergistic effects;
5. Head-to-head comparisons among modalities (e.g., mind–body vs. aerobic vs. multimodal) and intensity levels, with domain-specific cognitive endpoints.

Such advances will refine evidence-based guidelines and optimize exercise as a scalable, equitable strategy for AD prevention and care.

## Data Availability

The original data presented in the study are openly available in PubMed at https://pubmed.ncbi.nlm.nih.gov/.

## Abbreviations

3xTg	Triple-transgenic
Ab	Amyloid-beta
AD	Alzheimer’s disease
ADAS-Cog	Alzheimer’s Disease Assessment Scale–Cognitive subscale
AMPK	Adenosine monophosphate-activated protein kinase
*APOE*	Apolipoprotein E
*APOE* ε 4	Apolipoprotein E gene, epsilon 4 variant
APP/PS1	Amyloid precursor protein/presenilin-1
AQP4	Aquaporin-4
ARIAs	Amyloid-related imaging abnormalities
A^+^T^+^	Amyloid-positive/tau-positive
BBB	Blood–brain barrier
BDNF	Brain-derived neurotrophic factor
BMI	Body mass index
CNS	Central nervous system
CSF	Cerebrospinal fluid
CTBS	Cathepsin B
CVD	Cardiovascular disease
D-Gal	D-galactose
ER	Endoplasmic reticulum
FC	Frontal cortex
FNDC5	Fibronectin type III domain-containing protein 5
GSK-3β	Glycogen synthase kinase-3 beta
Hipp	Hippocampus
HIIT	High-intensity interval training
IGF-1	Insulin-like growth factor-1
IL-1b	Interleukin-1 beta
IL-6	Interleukin-6
IL-10	Interleukin-10
ISF	Interstitial fluid
LDL	Low-density lipoprotein
MCI	Mild cognitive impairment
MGBA	Microbiota–gut–brain axis
MMSE	Mini-mental state examination
MoCA	Montreal cognitive assessment
MR	Magnetic resonance imaging
MTCC	Mindful Tai Chi Chuan
NDEVs	Neuron-derived extracellular vesicles
NFT	Neurofibrillary tangle
NF-κB	Nuclear factor, kappa-light-chain-enhancer of activated B cell
NGF	Nerve growth factor
NPI	Neuropsychiatric inventory
NPS	Neuropsychiatric symptoms
NRF-1	Nuclear respiratory factor 1
Nrf2	Nuclear factor erythroid 2-related factor 2
NT3	Neurotrophin-3
PGC-1a	Peroxisome proliferator-activated receptor gamma coactivator 1-alpha
p-tau	Phosphorylated tau
proBDNF	Precursor brain-derived neurotrophic factor
QALY	Quality-adjusted life year
RAVLT	Rey Auditory Verbal Learning Test
RCT	Randomized controlled trial
ROS	Reactive oxygen species
SCFA	Short-chain fatty acid
SPPB	Short physical performance battery
STZ	Streptozotocin
t-tau	Total tau
TGF-β	Transforming growth factor-beta
TMT	Trail making test
TNF-a	Tumor necrosis factor-alpha
Trk	Tropomyosin receptor kinase
TrkB	Tropomyosin receptor kinase B
VEGF	Vascular endothelial growth factor
